# The association between cardiovascular medication use and survival in patients with advanced pancreatic adenocarcinoma

**DOI:** 10.1177/17588359261449094

**Published:** 2026-05-06

**Authors:** Michail N. Mavros, Farah Ladak, Kelvin K. W. Chan, Lena Nguyen, Natalie G. Coburn, Michael J. Raphael

**Affiliations:** Department of Surgery, Johns Hopkins University School of Medicine, Baltimore, MD, USA; Department of Surgery, University of Toronto, Toronto, ON, Canada; Division of Surgical Oncology, Odette Cancer Centre-Sunnybrook Health Sciences Centre, University of Toronto, Toronto, ON, Canada; Faculty of Medicine, University of Toronto, Toronto, ON, Canada; Department of Medical Oncology, Odette Cancer Centre, Sunnybrook Health Sciences Centre, Toronto, ON, Canada; ICES, Toronto, ON, Canada; ICES, Toronto, ON, Canada; Department of Surgery, University of Toronto, Toronto, ON, Canada; Division of Surgical Oncology, Odette Cancer Centre-Sunnybrook Health Sciences Centre, University of Toronto, Toronto, ON, Canada; ICES, Toronto, ON, Canada; Faculty of Medicine, University of Toronto, Toronto, ON, Canada; Department of Medical Oncology, Odette Cancer Centre, Sunnybrook Health Sciences Centre, Toronto, ON M4N 3M5, Canada; ICES, Toronto, ON, Canada

**Keywords:** combination chemotherapy, Cox proportional hazards model, drug repurposing, outcomes research, pancreatic cancer

## Abstract

**Background:**

Advanced pancreatic ductal adenocarcinoma (PDAC) has a poor prognosis and limited therapeutic options. Drug repurposing offers a cost-effective strategy to identify agents with potential synergistic benefits and known safety profiles. Preclinical and retrospective studies suggest that renin–angiotensin–aldosterone system inhibitors (RAASi) and statins may enhance chemotherapy efficacy by modulating the tumor microenvironment and oncogenic signaling.

**Objectives:**

To explore associations between cumulative RAASi and/or statin use and overall survival (OS) in elderly patients with advanced PDAC receiving first-line chemotherapy, as hypothesis-generating adjunct exposures.

**Methods:**

We analyzed a population-based registry using linked administrative databases from Ontario, Canada, identifying patients aged ⩾67 years with advanced PDAC who received first-line gemcitabine/nab-paclitaxel or mFOLFIRINOX between 2015 and 2024. The primary outcome was OS. Associations between cumulative RAASi or statin exposure and OS were assessed using multivariable Cox proportional hazards models, treating cumulative drug exposure as a time-varying covariate to mitigate immortal time bias.

**Results:**

Among 3226 eligible patients, the median age was 73 years, and 44% were female. Forty-one percent received both RAASi and statins, 17% statins alone, 14% RAASi alone, and 28% neither. Median OS was 7.9 months across groups. On multivariable analysis, neither cumulative RAASi (hazard ratio (HR) 1.00, 95% CI 0.98–1.02) nor statin exposure (HR 1.00, 95% CI 0.98–1.02) was associated with improved survival.

**Conclusion:**

In this large cohort of advanced PDAC patients undergoing first-line chemotherapy, real-world, non-targeted RAASi and statin use was not associated with improved OS. These findings highlight the need for rigorously designed prospective studies to validate repurposed drug candidates before clinical adoption.

**Design:**

Retrospective population-based cohort study.

## Plain language summary

Do blood pressure and cholesterol medications improve survival in older patients with advanced pancreatic cancer receiving chemotherapy?

**Why was this study done?** Pancreatic cancer is one of the hardest cancers to treat. Most patients are diagnosed at a late stage, when surgery is no longer an option, and even with chemotherapy survival is typically measured in months. Because developing new cancer drugs is slow and expensive, researchers have been asking whether medications people already take for other conditions, like blood pressure drugs or statins, might also help fight cancer. Both are cheap, widely available, and their side effects are well understood, which makes them appealing to study. What did this study do? We looked at health records from over 3,000 patients aged 67 and older in Ontario, Canada, who were treated for advanced pancreatic cancer between 2015 and 2024. All patients received standard chemotherapy. We compared survival between those who were also taking blood pressure medications, statins, both, or neither. What did this study find? No association was found between taking these medications and survival. Median survival was about 7.9 months across all groups, regardless of which medications patients were on. What do these findings mean? Taking blood pressure or cholesterol medications alongside chemotherapy does not appear to be associated with better survival in this population. While earlier, smaller studies hinted at a possible benefit, this larger real-world analysis did not confirm it. Carefully designed clinical trials, where patients are randomly assigned to receive these drugs or not, will be needed to settle the question.

## Introduction

Metastatic pancreatic adenocarcinoma is a disease with a poor prognosis and few treatment options. In the past two decades, only three new medications (and their combinations) have shown an overall survival benefit for the treatment of metastatic pancreatic ductal adenocarcinoma (PDAC): Erlotinib, Nab-Paclitaxel, and Nano-Liposomal Irinotecan. These medications improve overall survival in metastatic PDAC by only 2 weeks, 1.8 months, and 1.9 months, respectively.^1–3^ Owing to a lack of effective treatment options for PDAC, the median overall survival for patients who are well enough to receive first-line chemotherapy is only 7.7 months.^4^

The traditional cancer drug development pathway is slow, costly, and has a high translational failure rate.^5^ Among all cancer drugs tested in phase I clinical trials, only 3.4% eventually obtain FDA approval.^6,7^ Among those drugs that do obtain FDA approval, the median time from start of research and development to drug approval is 7.3 years (range, 5.8–15.2), and the median cost is $648 million USD (range, 157–1951 million).^8^ Unfortunately, most of these medications offer only modest benefits to patients. A review of all FDA-approved cancer medications between 2002 and 2014 identified median gains in overall survival of just 2.1 months.^9^ This is particularly concerning since the average price of a novel anticancer drug routinely exceeds $100,000 USD per year.^10^

In this context, drug repurposing represents a drug development strategy that may identify new, low-cost, synergistic treatments with already proven safety profiles.^11^ From a drug development perspective, repurposed drugs already have well-described pharmacodynamics, pharmacokinetics, and toxicity profiles.^12^ From a systems perspective, repurposed drugs are generally widely available and inexpensive, particularly if generic options already exist.^13^

Renin–angiotensin–aldosterone system inhibitors (RAASi) and statins are two classes of medications with substantial preclinical rationale that they may have benefit as repurposed drugs to improve outcomes for patients with advanced PDAC. The local pancreatic cancer microenvironment contains physical barriers to cancer treatment penetration, including a dense desmoplastic stroma.^14,15^ Preclinical research has shown that RAASi can decrease collagen to enhance the intratumoral concentration of nanotherapeutics and decrease hyaluronan to enhance chemotherapeutic efficacy.^16,17^ Similarly, statins have been hypothesized to improve cancer therapy efficacy through modulation of signal transduction, cell proliferation, differentiation, angiogenesis, and apoptosis.^18–20^ Moreover, statins have been shown to inhibit mutant KRAS and p53 and degrade mutant p53, resulting in cycle arrest and apoptosis in cancer cells.^21,22^

Therefore, the primary objective of this study is to evaluate potential associations between RAASi and statin use and survival outcomes in patients with advanced pancreatic cancer receiving first-line chemotherapy, within the scope of a retrospective observational design.

## Methods

### Study design

Using data linked from prospectively maintained administrative databases stored at ICES (Ontario, Canada), we conducted a population-based, retrospective cohort study of all incident cases of patients with PDAC who received treatment with first-line combination chemotherapy (gemcitabine-nab paclitaxel or mFOLFIRINOX) in Ontario, Canada between 2015 and 2024. Ontario has a population of 14.5 million people, representing nearly 40% of the Canadian population. Canada has a single-payer universal health insurance system with an associated comprehensive, mandatory healthcare administrative database collection. This study was designed, analyzed, and reported in accordance with the Strengthening the Reporting of Observational Studies in Epidemiology statement for cohort studies.^23^

### Data sources

The Ontario Cancer Registry (OCR) includes all patients with a cancer diagnosis (excluding non-melanoma skin cancer) in Ontario.^24^ The Registered Persons Database (RPDB) contains vital status and demographic data. Information regarding health services is included in the Canadian Institute of Health Information Discharge Abstract Database (CIHI-DAD), the National Ambulatory Care Reporting System, and the Ontario Health Insurance Plan (OHIP) Claims Database for billing from healthcare providers. The Cancer Activity Level Reporting (ALR) database includes chemotherapeutics and medications administered to cancer patients. The New Drug Funding Program (NDFP) database includes information on all approved injectable cancer medicines in Ontario, and the Ontario Drug Benefit (ODB) database includes information on outpatient drug prescriptions available to all patients aged 65 years and older. These datasets were linked using unique encoded identifiers and analyzed at ICES.

### Study population and cohort

Patients with a new diagnosis of pancreatic adenocarcinoma who received treatment with first-line combination chemotherapy (gemcitabine-nab paclitaxel or mFOLFIRINOX) over 2015–2024 were identified. Patients were excluded if they died before or on the date of diagnosis or were aged < 18 years at the time of diagnosis. Patients were eligible for inclusion in this study if they received at least one dose of first-line gemcitabine and nab-paclitaxel, or FOLFIRINOX, for locally advanced, unresectable, or metastatic pancreatic adenocarcinoma between 2015 and 2024. Patients were identified using the NDFP database, which covers the cost of all approved injectable cancer medicines in Ontario. Cases identified in the NDFP databases were deterministically linked by using unique, encrypted patient identifiers to administrative healthcare databases housed at ICES to identify baseline characteristics and survival outcomes. Only patients aged 67 years or older at the time of index treatment were included, given that data on outpatient medications (ODB) were available only for patients 65 years and older, and the lookback window was 2 years.

### Outcomes measure

The primary outcome was overall survival, defined as the time from index treatment until death (from any cause) or end of follow-up. Patients were followed from the date of index treatment until the date of death or May 31, 2025, up to a maximum of 5 years (at which point they were censored).

### Covariates

Demographic covariates included age (on the date of first treatment) and sex. Socioeconomic covariates included rurality (determined with the postal code of residence and defined as the Rurality Index of Ontario ⩾ 40) and income quintile (captured as the median income of a patient’s postal code of residence using national census data). Comorbidity burden was measured using the Charlson Comorbidity Index, which was calculated based on discharge claims data during the 2 years preceding the date of cancer diagnosis (categories: 0 vs 1 vs ⩾2). Eastern Cooperative Oncology Group (ECOG) Performance Status was obtained from NDFP. Treatment-related covariates included a history of prior pancreatic surgery or radiation, as well as systemic chemotherapy and/or clinical trial enrollment. We also collected data on the type of systemic chemotherapy during the study, the number of cycles, and second-line chemotherapy.

### Statistical analysis

Descriptive analyses were used to define baseline characteristics and outcomes. Categorical variables were reported as absolute numbers (*n*) and proportions (%), and continuous variables as means with standard deviation (SD) or medians with interquartile range (IQR), as appropriate. Chi-square tests compared categorical variables, while the Student’s *t*-test or Kruskal–Wallis test compared continuous variables, as appropriate. Overall survival was estimated using the Kaplan–Meier method, calculated with the life-table method, and compared with the log-rank test.

The primary analysis evaluated the hypothesis that a longer duration of exposure to the repurposed drug (RAASi and/or statin) is associated with longer overall survival among patients undergoing first-line chemotherapy for advanced PDAC. We estimated the effects of cumulative duration of repurposed drug exposure on overall survival using multivariable Cox proportional hazard models. Covariates were selected *a priori* and included age, sex, Charlson Comorbidity Index, and initial chemotherapy type. Repurposed drug exposure was modeled as a time-varying covariate to account for immortal time bias and to avoid an exclusive user vs non-user comparison. At time 0 (index treatment), individuals were assigned a value representing their cumulative dose received in the 2 years prior to index. After this point, information on this exposure was updated in the model on a daily basis for each patient. As a result, this analysis does not simply evaluate exposure time vs exposure-free time, but rather, patients are compared at every moment in time based on their cumulative drug exposure value at that specific point in time. Regression coefficients were then transformed to model the cumulative effect of drug exposure over 6-month intervals, since the incremental benefit of a single day or single week of drug exposure is expected to be clinically insignificant. Sensitivity analyses were performed using the Elixhauser Comorbidity Index (categories: 0 vs 1 vs ⩾2) and the Johns Hopkins Aggregated Diagnosis Groups score (categories: 0–4 vs 5–9 vs 10–14 vs ⩾15) for comorbidity risk adjustment, instead of the Charlson Comorbidity Index. The results are reported as hazard ratios (HRs) with 95% confidence intervals (95% CIs).

Analyses were conducted on a complete case basis. All analyses were two-sided with statistical significance at *p* ⩽ 0.05. Analyses were conducted using SAS Enterprise Guide 8.3 (SAS Institute, Cary, NC, USA).

## Results

**Figure 1.**
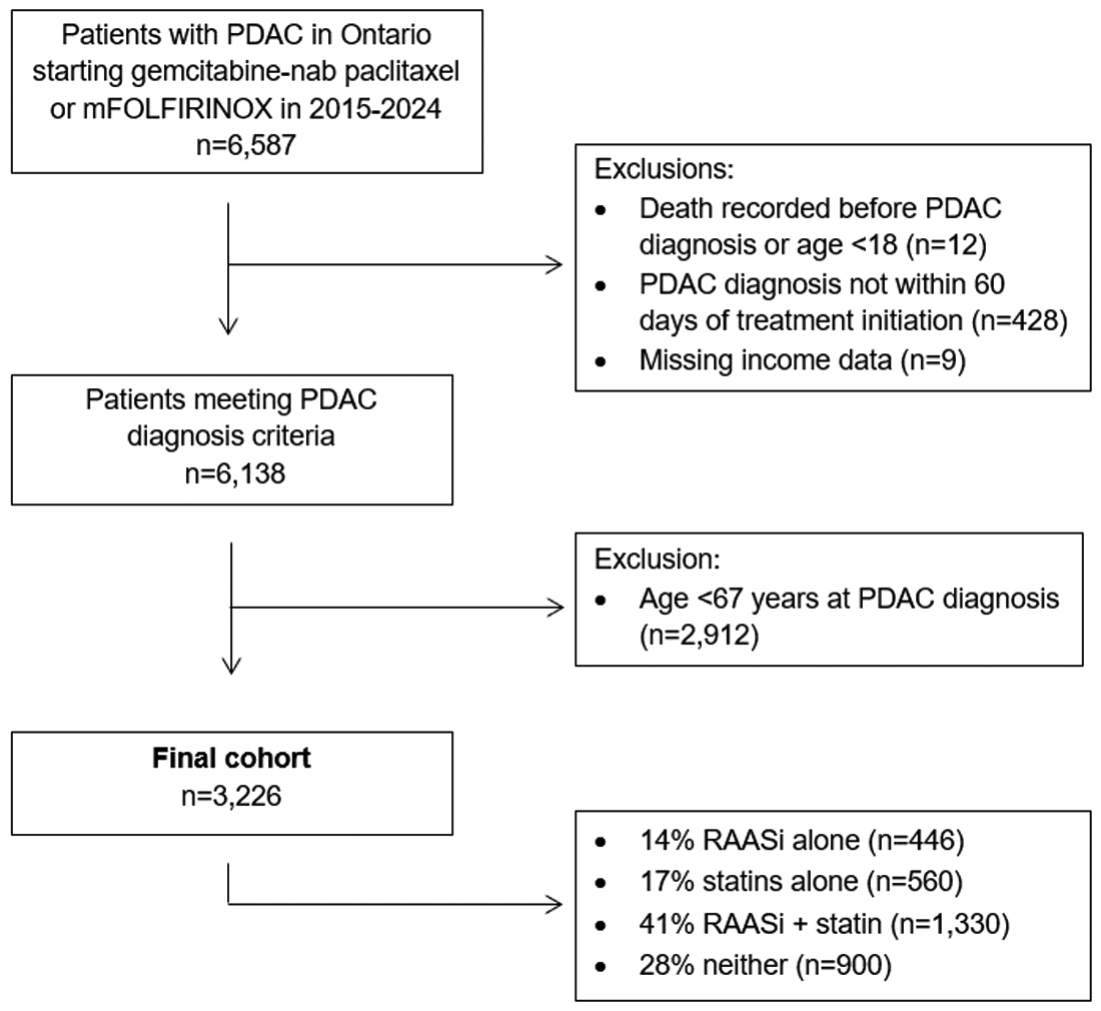
Flow chart of the cohort creation process.

Out of 6587 patients who received eligible systemic chemotherapy during the study period, 6138 patients met all inclusion and exclusion criteria, and full outpatient drug information was available on 3226 patients (Figure 1).

### Population characteristics

The median patient age was 73 years, and 44% were female (Table 1). About 41% of the patients received both RAASi and statin, 17% and 14% received statin or RAASi, respectively, and 28% received neither. ECOG score was missing for 1731 (53%) patients, and most of the remaining patients were ECOG 0-1 (1319, 41%). Roughly half (52%) of the patients had a primary PDAC of the pancreatic head, and 15% had undergone pancreatic resection (prior to experiencing cancer recurrence). Besides RAASi and/or statin, 25% of the patients also received beta-blockers, 30% calcium channel blockers, 23% diuretics, 34% metformin, 19% insulin, and 12% received thyroxine replacement. The population characteristics are presented in detail in Table 1.

**Table 1.** Baseline characteristics of included patients stratified by RAASi and statin use.

Characteristic	Total (*n* = 3226)	No RAASi/statin (*n* = 900)	RAASi (*n* = 446)	Statin (*n* = 560)	RAASi + statin (*n* = 1330)	*p*-Value
Age; years (median, IQR)	73 (70–77)	72 (69–76)	73 (70–78)	73 (70–78)	74 (70–78)	<0.0001
Sex (female)	1436 (44.4%)	494 (54.9%)	218 (48.9%)	255 (45.5%)	469 (35.3%)	<0.0001
Rurality	386 (11.9%)	111 (12.3%)	50 (11.2%)	70 (12.5%)	155 (11.7%)	0.89
Charlson Comorbidity Index						<0.0001
0	1011 (31.2%)	357 (39.7%)	159 (35.7%)	203 (36.3%)	292 (22.0%)	
1	557 (17.2%)	97 (10.8%)	92 (20.6%)	98 (17.5%)	270 (20.3%)	
2+	312 (9.6%)	29 (3.2%)	14 (3.1%)	47 (8.4%)	222 (16.7%)	
No inpatient data/missing	1356 (41.9%)	417 (46.3%)	181 (40.6%)	212 (37.9%)	546 (41.1%)	
ECOG status						0.39
0	368 (11.4%)	106 (11.8%)	52 (11.7%)	56 (10.0%)	154 (11.6%)	
1	951 (29.4%)	271 (30.1%)	143 (32.1%)	163 (29.1%)	374 (28.1%)	
2+	184 (5.7%)	46 (5.1%)	28 (6.3%)	23 (4.1%)	89 (6.7%)	
Missing	1731 (53.5%)	477 (53.0%)	223 (50.0%)	318 (56.8%)	713 (53.6%)	
Pancreatic tumor site						0.53
Head	1693 (52.3%)	472 (52.4%)	249 (55.8%)	290 (51.8%)	682 (51.3%)	
Body	570 (17.6%)	176 (19.6%)	71 (15.9%)	100 (17.9%)	223 (16.8%)	
Tail	456 (14.1%)	120 (13.3%)	59 (13.2%)	79 (14.1%)	198 (14.9%)	
Other	517 (16.0%)	132 (14.7%)	67 (15.0%)	91 (16.3%)	227 (17.1%)	
Prior pancreatic surgery	483 (14.9%)	140 (15.6%)	72 (16.1%)	83 (14.8%)	188 (14.1%)	0.69
Prior pancreatic radiation	185 (5.7%)	53 (5.9%)	22 (4.9%)	29 (5.2%)	81 (6.1%)	0.75
Time from diagnosis to treatment; days (median, IQR)	48 (29–97)	47 (28–100)	49 (30–98)	49 (29–101)	47 (29–90)	0.61
Doses of gemcitabine; median (IQR)	9 (4–16)	9 (4–17)	9 (5–15)	8 (3–17)	8 (3–15)	0.24
Doses of nab-paclitaxel; median (IQR)	9 (4–17)	9 (4–18)	9 (5–16)	9 (4–17)	9 (3–15)	0.32
Doses of oxaliplatin; median (IQR)	6 (3–12)	7 (3–12)	9 (4–13)	6 (3–11)	5 (2–11)	0.02
Doses of irinotecan; median (IQR)	6 (3–12)	7 (3–12)	8 (4–15)	6 (3–12)	5 (2–11)	0.04
Receipt of second-line gemcitabine	92 (2.8%)	35 (3.9%)	12 (2.7%)	17 (3.0%)	28 (2.1%)	0.10

ECOG, not otherwise specified; IQR, interquartile range; NA, not applicable; RAASi, renin–angiotensin–aldosterone system inhibitor.

Male patients were more likely to receive RAASi and/or statin (Table 1). Patients receiving both RAASi and statin had a higher prevalence of hypertension, congestive heart failure, and diabetes, and were more likely to also receive beta-blockers, calcium channel blockers, diuretics, insulin, and metformin (Supplemental Table 1).

### Outcomes

**Figure 2.**
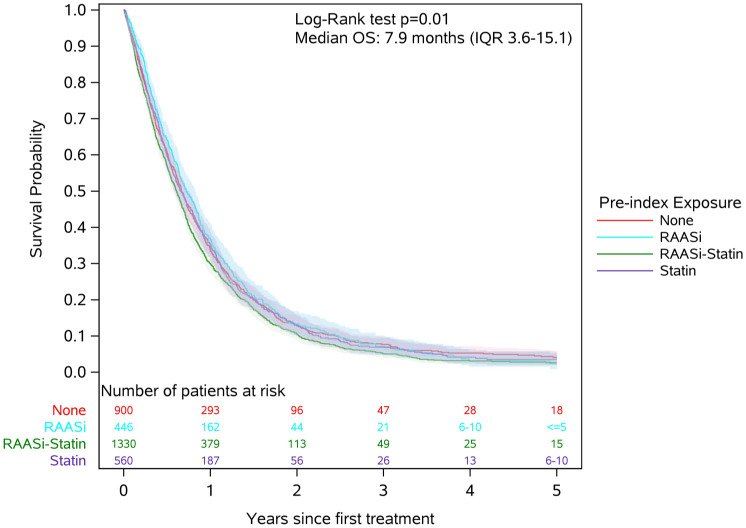
Unadjusted overall survival of patients with advanced PDAC receiving first-line systemic chemotherapy stratified by receipt of RAASi and/or statins. PDAC, pancreatic ductal adenocarcinoma; RAASi, renin–angiotensin–aldosterone system inhibitor.

The median overall survival for the entire cohort was 7.9 months (IQR 3.6–15.1), and 5-year survival was 1.4%. The unadjusted median overall survival was 7.4 months (IQR 3.3–14.3) among patients receiving both RAASi/statin, 8.0 months (IQR 3.7–14.9) among patients receiving statin only, 8.8 months (IQR 4.3–16.6) among patients receiving RAASi only, and 8.1 months (IQR 3.7–15.6) among patients receiving neither agent (Figure 2).

On multivariable analysis, the only variables independently associated with overall survival were receipt of FOLFIRINOX as the type of initial systemic chemotherapy (HR 0.78, IQR 0.72–0.85) and Charlson Comorbidity Index (score ⩾2 vs 0: HR 1.21, IQR 1.06–1.38). The cumulative dose of RAASi (HR = 1.00, IQR 0.98–1.02) or statin (HR = 1.00, IQR 0.98–1.02) was not independently associated with overall survival (Table 2). The sensitivity analyses utilizing the Elixhauser Comorbidity Index or the Johns Hopkins Aggregated Diagnosis Groups score yielded similar results (Supplemental Table 2).

**Table 2.** Multivariable Cox proportional hazards model for overall survival.

Characteristic	Hazards ratio	95% Confidence interval	*p*-Value
Age (per 10-year increment)	1.02	0.94–1.10	0.67
Male sex (reference: female)	0.96	0.90–1.04	0.32
Charlson Comorbidity Index (reference: 0)			
1	1.06	0.95–1.18	0.32
2+	1.21	1.06–1.38	0.004
No inpatient data/missing	0.95	0.87–1.04	0.25
mFOLFIRINOX as initial chemotherapy type (reference: gemcitabine/nab-paclitaxel)	0.78	0.72–0.85	<0.001
Cumulative statin dose (per 6-month use increment)	1.00	0.98–1.02	0.96
Cumulative RAASi dose (per 6-month use increment)	1.00	0.98–1.02	0.84

## Discussion

In this population-based cohort study, the use of RAASi and/or statins as adjuvants to first-line systemic chemotherapy was not associated with improved overall survival. The only factors independently associated with survival were the type of initial systemic chemotherapy regimen and comorbidity burden at baseline, consistent with prior literature.

While several retrospective studies have suggested potential antitumor effects of RAASi and statins across various malignancies, most have been limited by methodological biases that may inflate treatment effects. For example, multiple previous studies have identified that the putative benefits of statins and metformin on cancer outcomes seen in retrospective studies may be largely explained by immortal time bias.^25–30^ Immortal time refers to the period of follow-up during which, because of the exposure definition, the outcome of interest cannot occur. In studies comparing drug exposure (“users”) versus non-drug exposure (“non-users”), patients in the former group must necessarily be alive and event-free long enough to initiate the drug. This provides a guaranteed survival time that the “non-users” do not have. Thus, time-fixed drug exposures may artificially inflate the apparent survival benefit of drug exposure. Similarly, many prior analyses have been subject to “prevalent user” bias, which overestimates the treatment effect (prevalent users have by definition survived under treatment).^31^ To address these limitations, we modeled RAASi and statin exposure as a time-varying variable, with exposure information starting from 2 years prior to the diagnosis. This approach allowed for dynamic comparison of patients over time, based on their cumulative drug exposure rather than fixed binary categories.

Despite promising preclinical data suggesting that RAAS blockade may enhance drug delivery by modulating the tumor microenvironment, clinical results have been inconsistent. For instance, early single-institution studies of losartan combined with chemotherapy for locally advanced PDAC showed encouraging radiographic responses, but subsequent larger efforts, including two phase II comparative trials, did not replicate these findings.^32,33^ Similarly, although prior reports suggested RAASi might reduce platinum-induced neurotoxicity,^34–36^ this was not observed in our cohort, where patients (receiving mFOLFIRINOX) in both the RAASi and non-RAASi groups received a median of six doses of oxaliplatin. Our study adds to the published clinical evidence indicating no clinically significant improvement of survival in patients using RAASi and/or statins while receiving systemic chemotherapy for advanced PDAC.

In our sample, over two-thirds of included patients (aged at least 67 years) received RAASi and/or statins, consistent with population-level estimates in similar age groups.^37^ The median overall survival was roughly 8 months, which is also consistent with the literature for patients with advanced PDAC receiving first-line mFOLFIRINOX or gemcitabine nab-paclitaxel.^38^ Of note, the observed comorbidity burden, as captured by the Elixhauser Comorbidity Index, appeared lower than other series,^39^ possibly due to under-capture of certain chronic conditions. However, we performed sensitivity analyses using alternative indices (Charlson Comorbidity Index and the Johns Hopkins Aggregated Diagnosis Groups), which yielded consistent results, suggesting robustness of our findings.

Our study has several limitations. As a retrospective analysis of administrative datasets, residual confounding and misclassification are possible. On the other hand, the population-based design of this work is also a strength; the data encompass all patients in the jurisdiction with continuous data collection within a universal healthcare system and reflect real-world practices in both major academic centers and smaller community hospitals. Our study was limited to patients aged at least 67 years, to have complete outpatient prescription information. While this may limit generalizability to younger populations due to lower comorbidity burden and medication use, the mechanisms by which RAASi and statins may influence cancer outcomes are not inherently age-dependent. As these agents were evaluated as adjunct exposures in patients receiving standard oncologic care, the findings of this study should be interpreted as hypothesis-generating rather than definitive. Residual confounding, including confounding by indication, remains a concern despite multivariable adjustment and the use of comorbidity indices, as patients receiving RAASi or statins had higher rates of comorbidities and concurrent medication use. In addition, the absence of detailed information on dose intensity (e.g., high- versus low-intensity statins, ACE inhibitors versus ARBs), timing of initiation, and duration relative to cancer diagnosis and chemotherapy is a key limitation. Preclinical studies suggest that anticancer effects of statins and RAASi may be dose- and time-dependent, and this missing data may have attenuated detection of biologically relevant effects. Another important limitation is the use of the Elixhauser Comorbidity Index for risk adjustment. Although this index was derived from both inpatient and outpatient claims in our dataset, the overall comorbidity burden appeared lower than expected, suggesting possible under-capture of certain chronic conditions. Nevertheless, this limitation likely affected all groups equally and is therefore unlikely to have introduced meaningful bias. Moreover, missing data on ECOG performance status, a critical prognostic factor in pancreatic cancer, is an important limitation. Although sensitivity analyses were performed, incomplete ECOG information may have impacted treatment selection, survival outcomes, and residual confounding. Last, given the short median overall survival of the cohort (8 months) and the fact that treating oncologists had already deemed patients clinically fit to receive either mFOLFIRINOX or gemcitabine/nab-paclitaxel, much of the clinically relevant comorbidity adjustment was inherently accounted for in the treatment selection process.

Given the lack of observed survival benefit with RAASi or statin use in conjunction with first-line chemotherapy, these findings suggest that routine continuation or initiation of these agents solely for potential anticancer effects in advanced PDAC is unwarranted. However, the widespread use and favorable safety profile of these drugs make them appealing candidates for further investigation in combination with novel therapeutic strategies—particularly in well-designed, biomarker-driven prospective trials. Future studies should focus on identifying subgroups that may derive benefit, such as patients with specific metabolic or stromal features, and on clarifying mechanistic pathways through translational correlative work.

## Conclusion

In conclusion, in this large cohort of patients with advanced PDAC receiving first-line chemotherapy, the non-targeted, real-world use of RAASi and/or statins was not associated with improved overall survival after adjusting for treatment regimen and comorbidities. As these agents were evaluated as adjunct exposures rather than therapeutic interventions, the findings should be interpreted as hypothesis-generating rather than definitive. These findings underscore the importance of validating preclinical and retrospective signals through rigorously designed prospective studies before integrating such agents into oncologic care.

## Supplemental Material

sj-doc-1-tam-10.1177_17588359261449094 – Supplemental material for The association between cardiovascular medication use and survival in patients with advanced pancreatic adenocarcinoma Supplemental material, sj-doc-1-tam-10.1177_17588359261449094 for The association between cardiovascular medication use and survival in patients with advanced pancreatic adenocarcinoma by Michail N. Mavros, Farah Ladak, Kelvin K. W. Chan, Lena Nguyen, Natalie G. Coburn and Michael J. Raphael in Therapeutic Advances in Medical Oncology

sj-docx-2-tam-10.1177_17588359261449094 – Supplemental material for The association between cardiovascular medication use and survival in patients with advanced pancreatic adenocarcinoma Supplemental material, sj-docx-2-tam-10.1177_17588359261449094 for The association between cardiovascular medication use and survival in patients with advanced pancreatic adenocarcinoma by Michail N. Mavros, Farah Ladak, Kelvin K. W. Chan, Lena Nguyen, Natalie G. Coburn and Michael J. Raphael in Therapeutic Advances in Medical Oncology

## Declarations

**Ethics approval and consent to participate:** The study protocol was approved by the Research Ethics Board of the Sunnybrook Health Sciences Centre in Toronto, Ontario (Project ID# 6128, “Can we re-purpose existing medications to improve cancer outcomes in pancreatic cancer?”). The requirement for informed consent was waived due to the retrospective design of the study, posing minimal risk to participants.

**Consent for publication:** Not applicable.

**Availability of data and materials:** The data that support the findings of this study are not publicly available due to restrictions related to patient privacy and confidentiality. Aggregate data supporting the findings may be available from the corresponding author* upon reasonable request, subject to institutional approvals and applicable data use agreements.
