# Supplementary material for: The association between cardiovascular medication use and survival in patients with advanced pancreatic adenocarcinoma
Source: Ther Adv Med Oncol. 2026 May 6;18:17588359261449094. doi: 10.1177/17588359261449094 (PMC13161621; doi:10.1177/17588359261449094)
Supplement: sj-doc-1-tam-10.1177_17588359261449094 – Supplemental material for The association between cardiovascular medication use and survival in patients with advanced pancreatic adenocarcinoma [file sj-doc-1-tam-10.1177_17588359261449094.doc]

**SUPPLEMENTAL TABLES**

| **Supplemental Table 1.** Receipt of other medications among the groups. | | | | | | |
| --- | --- | --- | --- | --- | --- | --- |
| Characteristic | Total  (n=3,226) | No RAASi/Statin  (n=900) | RAASi  (n=446) | Statin  (n=560) | RAASi+Statin  (n=1,330) | p-value |
| Beta blocker | 800 (24.7%) | 95 (10.6%) | 92 (20.6%) | 128 (22.9%) | 485 (36.5%) | <0.001 |
| Calcium channel blocker | 957 (29.6%) | 111 (12.3%) | 180 (40.4%) | 128 (22.9%) | 538 (40.5%) | <0.001 |
| Diuretics | 747 (23.1%) | 113 (12.6%) | 123 (27.6%) | 95 (17.0%) | 416 (31.3%) | <0.001 |
| Insulin | 607 (18.8%) | 63 (7.0%) | 67 (15.0%) | 84 (15.0%) | 393 (29.5%) | <0.001 |
| Metformin | 1,083 (33.5%) | 112 (12.4%) | 113 (25.3%) | 173 (30.9%) | 685 (51.5%) | <0.001 |
| Proton pump inhibitors | 2,062 (63.7%) | 506 (56.2%) | 293 (65.7%) | 387 (69.1%) | 876 (65.9%) | <0.001 |
| H2 receptor blockers | 229 (7.1%) | 58 (6.4%) | 26 (5.8%) | 48 (8.6%) | 97 (7.3%) | 0.31 |

| **Supplemental** **Table 2a.** Sensitivity analyses of multivariable Cox proportional hazards model for overall survival using the Elixhauser Comobidity Index for comorbidity adjustment. | | | |
| --- | --- | --- | --- |
| Characteristic | Hazards Ratio | 95% Confidence Interval | p-value |
| Age (per 10-year increment) | 1.02 | 0.94-1.10 | 0.66 |
| Sex (male) | 0.96 | 0.89-1.03 | 0.24 |
| Elixhauser Comorbidity Index (reference: 0)  1  2+ | 0.87  1.12 | 0.80-0.95  1.02-1.23 | <.001  0.01 |
| mFOLFIRINOX as initial chemotherapy type | 0.78 | 0.71-0.84 | <.001 |
| Cumulative statin dose (per 6-month use increment) | 1.00 | 0.98-1.02 | 0.78 |
| Cumulative RAASi dose (per 6-month use increment) | 1.00 | 0.98-1.02 | 0.98 |

| **Supplemental** **Table 2b.** Sensitivity analyses of multivariable Cox proportional hazards model for overall survival using the Johns Hopkins Aggregated Diagnosis Groups (ACG) score for comorbidity adjustment. | | | |
| --- | --- | --- | --- |
| Characteristic | Hazards Ratio | 95% Confidence Interval | p-value |
| Age (per 10-year increment) | 1.01 | 0.93-1.09 | 0.82 |
| Sex (male) | 0.96 | 0.89-1.03 | 0.23 |
| ACG Comorbidity Score (reference: 0-4)  5-9  10-14  15+ | 1.00  0.98  1.12 | 0.86-1.17  0.84-1.14  0.92-1.35 | 0.95  0.79  0.27 |
| mFOLFIRINOX as initial chemotherapy type | 0.77 | 0.71-0.84 | <.001 |
| Cumulative statin dose (per 6-month use increment) | 1.00 | 0.98-1.02 | 0.89 |
| Cumulative RAASi dose (per 6-month use increment) | 1.01 | 0.99-1.02 | 0.56 |
